# Non-invasive quantitative imaging of hepatocellular carcinoma growth in mice by micro-CT using liver-targeted iodinated nano-emulsions

**DOI:** 10.1038/s41598-017-14270-7

**Published:** 2017-10-24

**Authors:** Nicolas Anton, Alexandru Parlog, Ghina Bou About, Mohamed F. Attia, Marie Wattenhofer-Donzé, Hugues Jacobs, Isabelle Goncalves, Eric Robinet, Tania Sorg, Thierry F. Vandamme

**Affiliations:** 1 University of Strasbourg, Faculty of Pharmacy, 74 route du Rhin 67401 Illkirch-Graffenstaden Cedex, Strasbourg, France; 2 CNRS UMR 7199, Laboratoire de Conception et Application de Molécules Bioactives, équipe de Pharmacie Biogalénique, 74 route du Rhin 67401 Illkirch-Graffenstaden Cedex, Strasbourg, France; 3 CELPHEDIA, PHENOMIN, Institut Clinique de la Souris, 1 rue Laurent Fries, 67404, Illkirch, France; 4 Centre National de la Recherche Scientifique, UMR7104, 1 rue Laurent Fries, 67404 Illkirch, Paris, France; 5 Institut National de la Sante et de la Recherche Médicale, U964, 1 rue Laurent Fries, 67404 Illkirch, Paris, France; 6 Université de Strasbourg, 1 rue Laurent Fries, 67404 Illkirch, Strasbourg, France; 7 National Research Center, P.O., 12622 Cairo, Egypt; 8 Present Address: Department of Bioengineering, Clemson University, 203 Rhodes Annex, Clemson, SC 29634 USA; 9 IHU-Strasbourg, Institute of Image-Guided Surgery, 67000 Strasbourg, France

## Abstract

Hepatocellular carcinoma (HCC) is the only cancer for which non-invasive diagnosis is recognized by international guidelines. Contrast agent free ultrasound imaging, computed tomography (CT) and/or magnetic resonance imaging are techniques used for early detection and confirmation. Clinical evidence depicts that CT is 30% less precise as compared to MRI for detection of small tumors. In our work, we have reported some novel tools that can enhance the sensitivity and precision of CT applied to preclinical research (micro-CT). Our system, containing non-toxic nano-droplets loaded with iodine has high contrasting properties, liver and hepatocyte specificity and strong liver persistence. Micro-CT was performed on HCC model implanted in nude mice by intrahepatic injection. Contrast agent was administrated intravenously. This method allows an unprecedented high precision of detection, quantitative measurement of tumor volume and quantitative follow-up of the tumor development.

## Introduction

Hepatocellular carcinoma (HCC) is the 5^th^ most common cancer globally, representing 6% of all cancers, with a high mortality to incidence ratio and a unique geographic^1,2^. Common risk factors for HCC are hepatitis B or C and non-viral liver cirrhosis^3–5^. However, its association with Hepatitis B or C and other risk factors is mainly governed by geographical location. This argument is supported by the prevalence data of this disease as there are 15 cases per 100,000 persons in China and sub Saharan African populations as compared to 3 cases per 100,000 persons in North America and Western Europe. Since 2001, non-invasive diagnosis of HCC was accepted globally as a replacement to conventional biopsy method and till today it is only tumor for which noninvasive diagnosis is recognized as diagnostic tool. However, clinical guidelines are still discussing this point^6^. In some cases well-defined non-invasive diagnostics of HCC can be established without biopsy. These cases are mainly described as the hypervascularity in the arterial phase with washout in the portal, or, when clinicians observe 3 min-delayed phases in a nodule higher than 1 cm in diameter. On the other hand, although biopsy can pose many serious risks such as coagulopathies, susceptibility to infection and risk of spreading HCC through the needle tract, it is still advocated as confirmatory tool for all indeterminate nodules on imaging work-up by contrast-enhanced scans^7,8^.

When HCC is diagnosed at an advanced stage, the prognostic indicators suggests that for 95% of patients survival is not more than 5 years^9^. On the other hand, early diagnosis of HCC by biomedical imaging allows improving prognosis, especially true for population with the risk factors discussed above^9–11^.

Noninvasive imaging of HCC is generally based on enhancement of patterns in the arterial phase, using extracellular contrast agent, or a washout pattern in the portal venous and equilibrium phases, potentially using a post-vascular contrast agent^12^. International guidelines approved several complementary imaging techniques for the clinical surveillance and diagnosis of HCC, namely ultrasound (US), computed tomography (CT) and magnetic resonance imaging (MRI). All international guidelines approved that the surveillance tests are performed though ultrasonography, whereas four-phase CT and dynamic contrast MRI are performed for first-line diagnosis^13,14^ (according to guidelines of the American Association for the Study of Liver Diseases (AASLD), or of the European Association for the Study of the Liver (EASL)). It is noteworthy that contrast enhanced ultrasound (CEUS) imaging is thus relegated at second/third-line diagnosis, and positron emission tomography (PET)-CT is not included in the guidelines for HCC diagnosis. This statement not only emphasizes the crucial roles and complementarity of CT and MRI imaging for HCC diagnosis, but also calls for the development of advanced contrast agents.

Contrast agents used for HCC diagnosis are either *(i)* extracellular contrast agents; iodine-based for CT and gadolinium chelate-based for MRI, or *(ii)* post-vascular phase agents that are either taken-up by Kupffer and/or reticuloendothelial cells, or by hepatocytes. Imaging Kupffer cells is performed by CEUS (using *Sonazoid* ^TM^ (GE Healthcare, UK), *Levovist* ^TM^ (Bayer Schering, Germany)) or T_2_-weighted MRI contrast agents, exhibiting a very stable phase, and allows identifying hepatocarcinogenesis from dysplastic nodules^15,16^, whereas hepatocyte imaging is performed by T_1_-weigthed MRI imaging agents (using gadoxetic acid, Gd-EOB-DTPA, gadoxetate disodium, *Primovist* ^TM^/*Eovist* ^TM^ (Bayer Schering, Germany), or Gadobenate dimeglumine *Gd-BOPTA*, *Multihance* ^TM^ (Bracco, Italy).

From literature review, MRI imaging stands out as the gold standard for early diagnosis, selective and functional imagining of HCC. Moreover, if this diagnosis approach is comparable with the one undertaken for the detection of liver metastasis^17^, the strength of MRI lies in several physical techniques giving complementary information, like *(i)* fat-suppressed T_2_-weihted imaged for determining the tumor location, *(ii)* contrast-enhanced T_1_-weighted images that show a ring enhancement around the tumor, and *(iii)* confirmed by diffusion-weighted sequence. Overall the clinic experience reported that MRI modality allows identifying metastasis unseen by US, CT or PET-CT^17^. Particularly, compared to contrast-enhanced MRI, the CT was recognized around 30% less precise^18–21^. This is mainly because of very high (50%) accumulation efficiency of gadoxetate disodium in hepatocytes. This reveals that the two key-points of the imaging efficiency of contrast agent depend on its *(i)* liver specificity and *(ii)* hepatocyte-specificity.

In the general context of HCC or metastasis imaging in liver, the limitation of CT comes from the limitation of the *in vivo* properties of the available CT contrast agents. MRI techniques proposed several experimental possibilities like which T_1_-, T_2_-, diffusion-weighting sequences (among others), with and without contrast agents, that enable several types of complementary information on the liver pattern. In contrast, imaging soft tissues with the CT modality necessary involves using a contrast agent. The pattern imaging only depends (and linearly) on the concentration of contrast agent in the organ, as well as the difference in contrast between the tumor and liver. Since, the resolution and sensitivity of tumor detection is directly related to the tumor-to-liver contrast, as demonstrated in literature for hypo-vascular tumor, and *a fortiori* for post-vascular imaging^22^. Recent preclinical studies conducted on CT, T_1_- and T_2_-weighted imaging, emphasized that a lower detection limit of hepatic lesion is around 2–3 mm^17,23–25^. Therefore, the key factors that can improve the sensitivity of the tumor detection by CT may be linked to the concentration ofX-ray contrasting atoms (like iodine) in hepatocyte.

This is precisely the purpose of the present study, where we describe new contrast-enhanced solutions that significantly improve the efficiency of the micro-CT imaging, for the detection of hypo-contrasted liver lesions in the post-vascular phase. It is important to note that the present study was performed with a micro-CT scanner, which has important difference with clinical scanners. The transposition to clinical CT of the contrast agent described is a realistic potential outcome. In fact, besides the structural difference, when clinic CT have a helical configuration with large focal spot and micro-CT reaches resolutions as small as 100 μm (voxel side), their fundamental difference actually comes in their applications. This is due to the fact that available contrast agents used in clinic are small hydrophilic iodinated molecules, which are excreted by the kidney only a few minutes after i.v. administration. Therefore, considerable investments were made by industry to adapt the scanners to the *in vivo* properties of the contrast agents to reduce the acquisition time. Such clinic contrast agents circulate in blood pool and do not accumulate in soft tissues. Due to the fast blood clearance (in the order of the acquisition time of micro-CT) the use of clinic contrast agent is not compatible with preclinical scanners. A false solution would be high doses or multiple administrations, but results in significant side effects, like nephropathy, acute kidney injury, or renal failure. On the other hand another family of contrast agents was developed^26^ in the form of nanoparticles, higher than the glomerular size pore of 10 nm, thus increasing the circulation time in blood pool and making possible the specific accumulation in soft tissues and organs, finally compatible with micro-CT. It follows therefrom that, even if the clinical CT and micro-CT scanners are different, and even if the clinical contrast agents are not usable in micro-CT scanner for pharmacokinetics reasons, the opposite is not true and technologies developed in for micro-CT are in principle transposable in clinic. The limitation that remains noteworthy will be the regulatory aspects and the regulatory validation of the formulation developed.

The idea lies in using hepatocyte selective tri-iodinated molecules, *i.e*. 2-3-5-triiodo α-tocopheryl^27^, formulated as non-toxic PEGylated nano-emulsions to prevent renal clearance, and to ensure a prolonged circulation time in blood pool with half-life in blood around 9 hours. One important particularity of this formulation is its total innocuousness since based on α-tocopherol (vitamin E), and the outstanding persistence in of the contrast enhancement in liver higher than 3 months after a single injection, much better than similar nanoparticles based formulations^28^. This study will focus on the imaging a humanized orthotopic model of HCC (based on the injection of luciferase expressing human hepatoma Huh-7 (Huh-7-Luc)), and follow the tumor apparition and growth after a single i.v. administration of CT contrast agent. Objectives of the current study are evaluating this innovative CT contrast agent for non-invasive HCC diagnosis in mice. Based on preclinical imaging, we will explore the feasibility in the detection and follow-up of HCC tumor implanted into the liver, comparing to the other modalities reported in literature for MRI^23–25,29^, and BLI^30–32^. Comparison between the different modalities is performed through the resolution of the nodule detection and the precision over a population studied, taken as absolute result. This allowed, as it is commonly done in literature, to compare the effectiveness and sensitivity of the imaging techniques. In addition, for all animal followed in micro-CT, we performed US acquisitions and analysis, to compare the modalities and to show the extent of the precision gained by the nano-emulsions technology.

## Results

### Iodinated nano-emulsions

**Figure 1:**
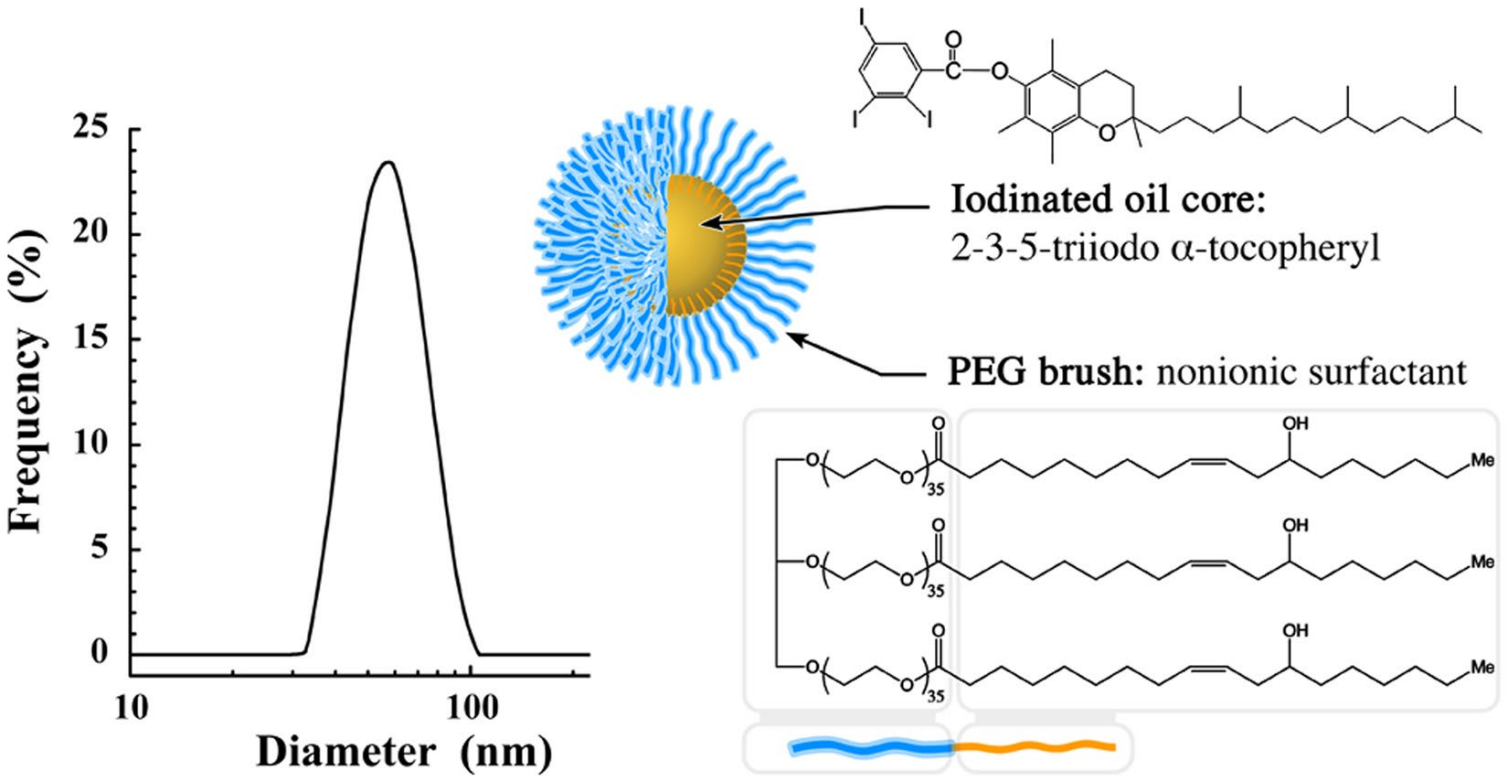
Size distribution of the iodinated nano-emulsions determined by dynamic light scattering, and schematic representation of a nano-emulsions droplet composed of 2-3-5-triiodo α-tocopheryl as oily core and Kolliphor ELP^®^ as stabilizing agent (the blue part is the PEG polar head, and orange part is the lipophilic moiety).

Size distribution (Fig. 1) of nano-emulsions is centered to 60 nm with a polydispersity index (PDI) equal to 0.08 testifying the sample monodispersity. Iodinated oil core consists of 2-3-5-triiodo α-tocopheryl (see Fig. 1) composed of 42 wt.% of iodine, formulated in the form of nano-droplets decorated with polyethylene glycol (PEG) (Mw^PEG^ *ca*. 1500 g/mol) as polar moiety of the stabilizing agent (Kolliphor ELP^®^, where chemical structure shows the PEG moiety in blue and aliphatic one in orange).

### Visualization and follow-up of HCC tumor growth in liver

**Figure 2:**
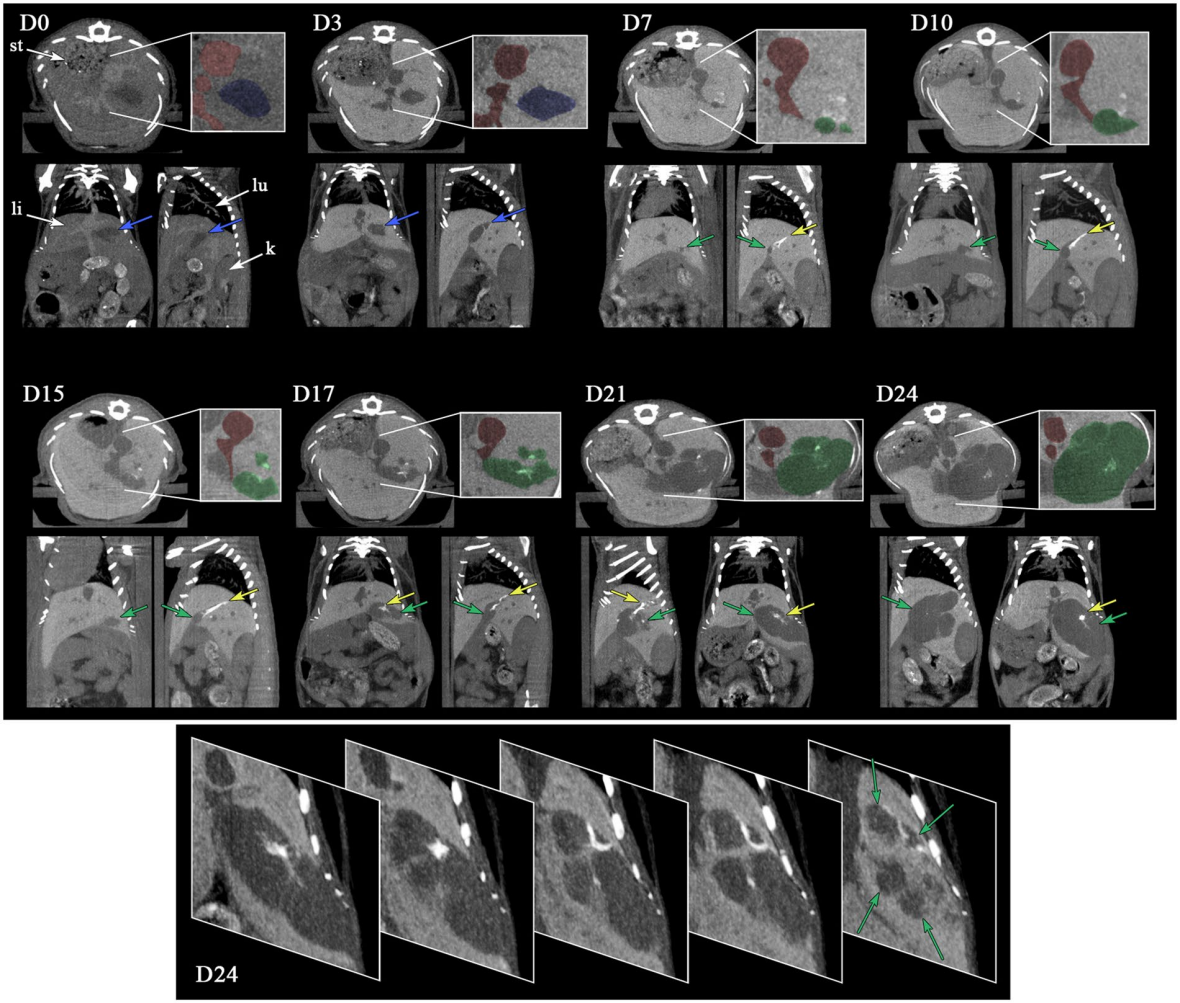
Longitudinal study of the tumor growth in liver of nude mouse (for a representative mouse M1) after intrahepatic injection of Huh-7 cells, and i.v. administration of 2-3-5-triiodo α-tocopheryl nano-emulsions at D0. Acquisitions were performed by micro-computed tomography (micro-CT), and the pictures report maximum intensity projection of transverse section, sagittal and coronal section of the liver, for 0, 3, 7, 10, 15, 17, 21 and 24 days after injection of the cells and iodinated nano-emulsions as contrast agent. At D0, are indicated in the figure the localizations of the stomach (st), liver (li), lung (lu) and kidney (k). Insets show details with colored filters that guide the eye to differentiate the vascularization (in red), cell injection cavity (in blue), and tumor (in green), completed by arrows in the other views. Yellow arrows point out the hyper-contrast zones that appear during the tumor growth. The bottom part, at D24, shows transverse sections of tumor for different depths, emphasizing the different tumor nodules with green arrows.

Implantation of Huh-7 was immediately followed by the i.v. administration of 2-3-5-triiodo α-tocopheryl nano-emulsions, noted “day zero” (D0) in the timeline. This experiment was conducted on a group of 15 mice and intrahepatic tumor engraftments were clearly visible in 10 mice. The pharmacokinetics of these nano-emulsions in healthy mice showed a total blood clearance and maximum hepatic accumulation after 24 h^27^. Expectedly this is confirmed in orthotopic HCC tumor bearing mice, in which the vascular contrast is largely enhanced at D0 and the hepatic pattern (*i.e*. healthy tissue of liver) very contrasted in the post-vascular phase (firstly visible at D3). Figure 2 reports the micro-CT acquisition from D0 up to D24 for a representative mouse (M1), showing, for each time-point, the transverse section of liver, sagittal and coronal sections. For each time-point, an inset is focused on the transverse section to emphasize the different morphological details with a color code: blood compartment, venae cava in red; the initial cavity in liver corresponding to the injection of Huh-7 cells in culture medium in blue; and the location of HCC tumor once formed in green. At D0 the contrast agent is clearly located in the blood pool showing the irrigation of liver, disclosing the venae cava and other smaller veins (that appear brighter than liver tissues in the different views and highlighted in red). The location of Huh-7-cells (blue in the inset, also shown by the blue arrows in the sagittal and coronal views) is clearly visible as negative contrast, close to the venae cava exactly in the area where was performed the US guided cell implantation. At D3, the 2-3-5-triiodo α-tocopheryl is accumulated in the liver tissue (in hepatocytes^27^) that appears very bright, and confirmed the location of both liver irrigation (this time by negative contrast) and cell implantation region. Next, the cavity has resorbed at D7 along with the beginning of the HCC tumor growth (expectedly in the same location) indicated in green in inset and by green arrows. Surprisingly, a hyper-contrast appears in the close region around the HCC location, adopting a longitudinal shape (yellow arrow), and showing a value of the contrast enhancement of *ca*. 1140 HU, when the contrast-enhanced liver tissue is *ca*. 190 HU and bone *ca*. 1500 HU. In the whole longitudinal study herein presented, the pictures of D7 show HCC tumors having the smallest dimensions; we can detect two small nodules (Fig. 2, D7, inset indicated in green) measured at 1.0 mm and 0.6 mm. The nodules merged at D10 along with the tumor growth, and seem to emerge near the hyper-contrasted domain (yellow arrow). This is confirmed with D15 and up to D24, with a continuous and gradual increase of the tumor size, totally embedding the hyper-contrasted point. At the end of the period followed, HCC tumor is totally embedded within the liver, exhibiting several lobes developed around a hyper-contrasted region. On the details on the D24 tumor (bottom part), we can see (from right to left) the different nodules separated from bridges of healthy liver tissues, brighter, which gradually confine the contrast agents into small domain becoming hyper-contrasted.

### Histological analysis

**Figure 3:**
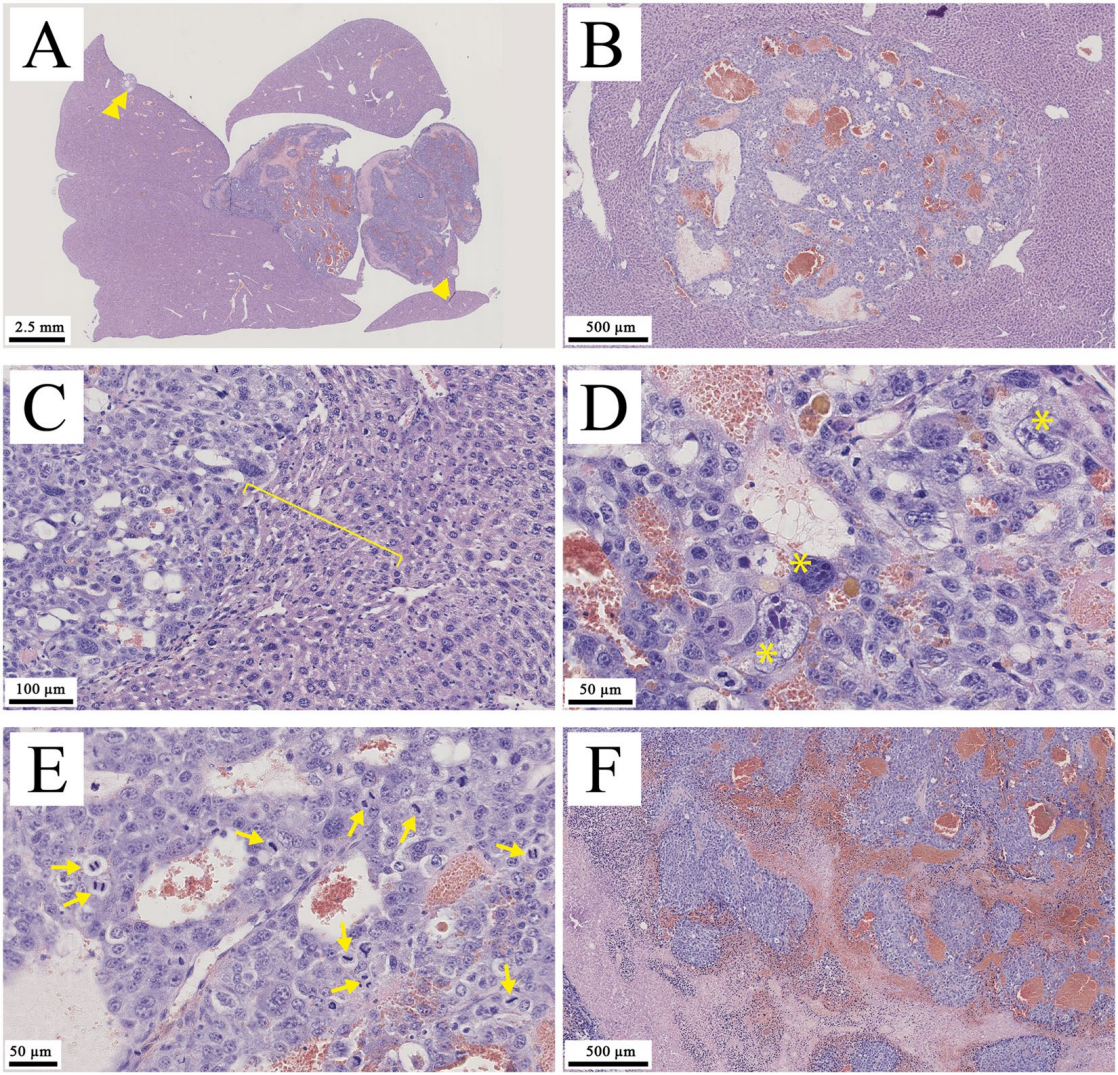
Hepatocellular carcinoma. (**A**) Externalized tumor. Note basophilic (purple-blue) and necrotic (pale pink) areas. Arrowheads point to small intra lobar tumors. (**B**) Intra lobar tumor. Note clear delimitation between tumor and normal tissue. Angiectasis is evident. (**C**) Tumor border. Note absence of capsule and compression of normal tissue (bracket). (**D** and **E**) Histological organization. Note that hepatocyte differentiation is maintained although some giant cells have developed (aster) and high mitotic rate (arrow). Normal histological organization is lost. (**F**) Necrosis and hemorrhage. H&E staining. Scale bares are indicated on pictures.

Examination performed on the mouse M1 are reported in Fig. 3, and confirm that the inclusion in liver observed above in Fig. 2 effectively corresponds to tumor tissue. On overall examination, hepatic tumors were well-delimited round basophilic tumors. Although not encapsulated, tumors constantly compressed normal hepatocytes at vicinity and a palisade was organized at border. Larger tumors migrated towards the outside of hepatic lobe. On histological aspects, tissue organization is lost, cystic dilatations and hemorrhages were frequent and necrosis foci were observed on larger tumor. No significant inflammation was visible. From cytological point of view, even though hepatic tumor cell were still differentiated, giant cells were frequent, and atypical nuclei were observed. Mitotic rate was high. Tumors were diagnosed as differentiated solid hepatocellular carcinoma.

### Quantification of X-ray attenuation

**Figure 4:**
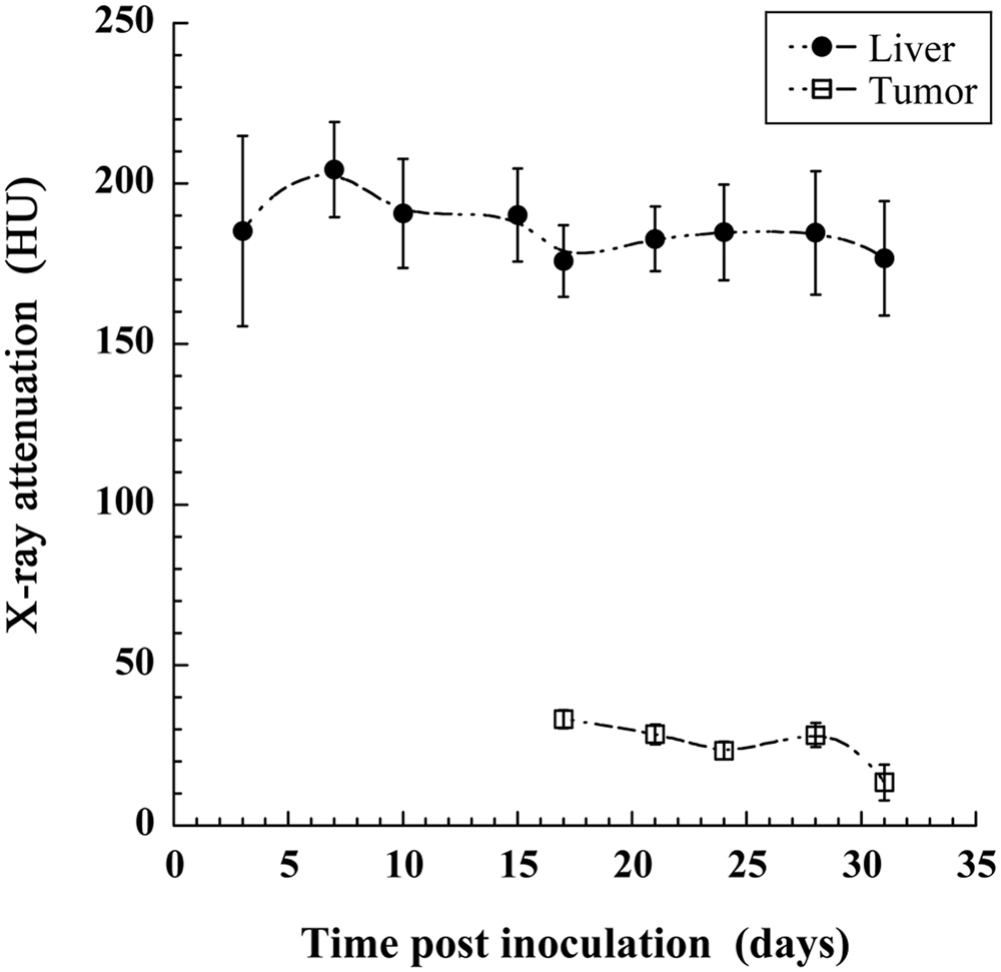
Longitudinal follow-up of the X-ray attenuation in liver and in the tumor region. Bars are the standard deviations (n = 3).

Another important possibility offered by X-ray imaging is the quantification of the concentration of contrast agent that has effectively reached the organ or region of interest. The actual huge liver specificity of 2-3-5-triiodo α-tocopheryl raises the question of the accumulation of the nano-emulsion also in the tumor region. If the signal in liver is huge compared to the one in the tumor, it is possible that nano-emulsions also accumulate in the tumor region but it is not visible on the intensity projections pictures. ROI are placed on the one hand in the liver tissues and on the other hand in the tumor region (excluding the hyper-signal region, yellow arrows in Fig. 2). Results are summarized in Fig. 4, and disclose high signal in liver, from 200 HU to 170 HU, and rather stable over the experiment period. In contrast, the tumor area appears non-contrasted, around and lower than 25 HU, and dropping close to the signal of attenuation of pure water (0 HU) at D31, evidencing the strong liver-to-tumor contrast enhancement. This result shows the possibility to delineate the tumor region with precision, to quantify and follow its volume.

### Quantification of the HCC tumor growth

**Figure 5:**
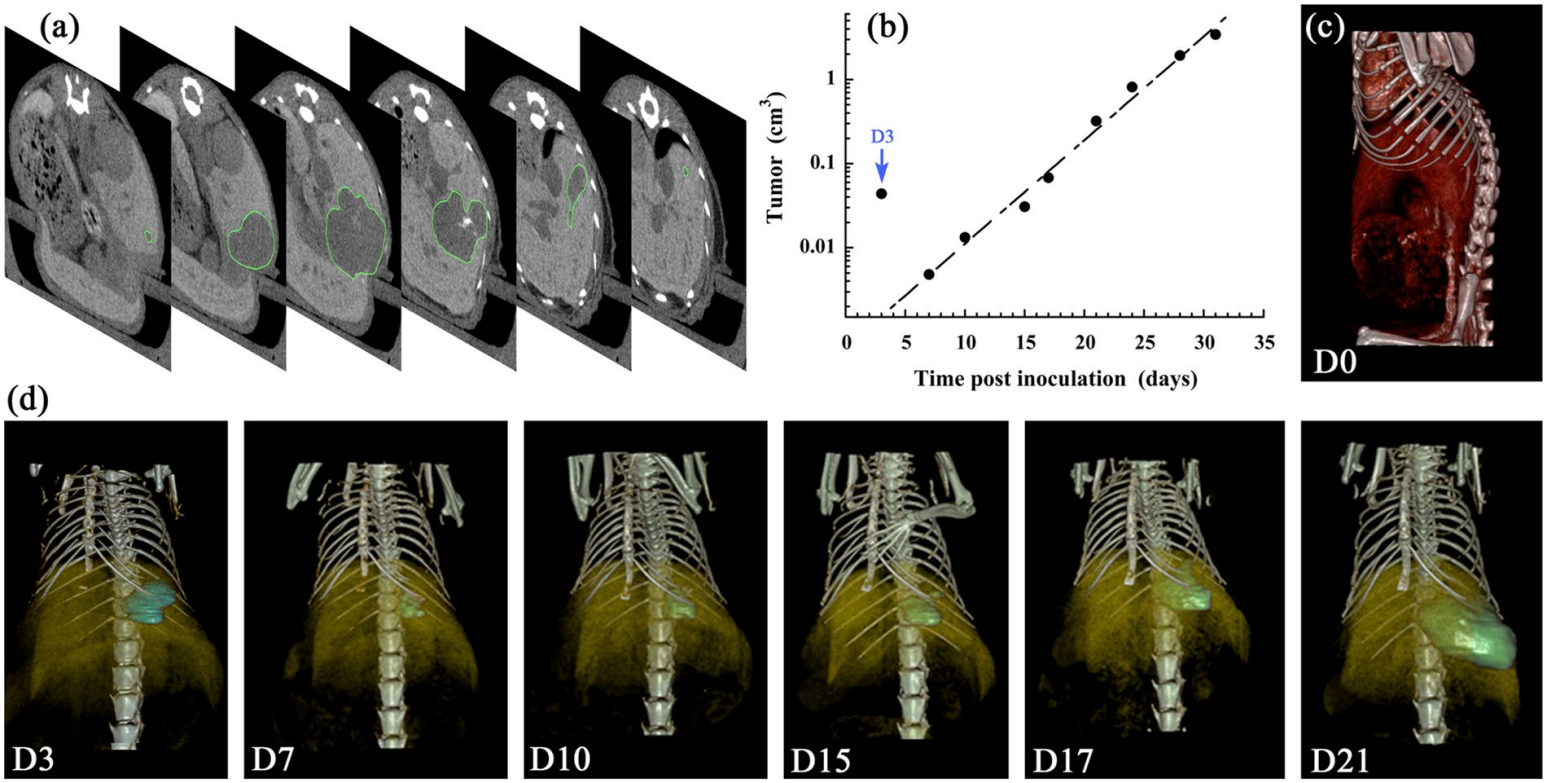
Quantitative measurement of the tumor volume (on a representative mouse M1), corresponding to the longitudinal study reported in Fig. 2, obtained by micro-CT, after intrahepatic injection of Huh-7 cells, and i.v. administration of 2-3-5-triiodo α-tocopheryl nano-emulsions at D0. (**a**) At D21, transverse slices of liver at different positions in the animal, in order to show the ROI corresponding to the tumor (green contour). (**b**) Tumor volume in function of the time post inoculation of Huh-7 cells (excepted D3, corresponding to the cavity of the cells injection). The data are fitted according to the tumor growth exponential model of Eq. 1 (excluding D3). (**c**,**d**) 3D volume rendering showing the blood compartment in red (D0), the liver in yellow (D3-D21), the injection cavity in blue (D3), and the tumor in green (D7-D21).

The quantification of the tumor growth corresponding to the Fig. 2 (*i.e*. for a representative subject M1) is reported in Fig. 5. This experiment has been reproduced on a whole group of 10 mice (M1 to M10), which is reported and discussed in the following section. The regions of interest (ROI) have been carefully placed on the different tumors of each specific time-point presented in Fig. 2 above. Figure 5a shows transverse sections of M1 at D21, at different locations in the mouse, on which we can see the ROI delimited by the green line. In that way the HCC tumor volume is measured with a high precision throughout the longitudinal study, reported in Fig. 5b, in good accordance with the exponential growth model^33^, widely used in literature to model tumor growth:

1 $$V(t)={V}_{0}{e}^{\lambda t}$$

where *V(t)* is the tumor volume at time *t*, *V*_0_ the tumor initial volume, and *λ* the growth constant. In the representative example shown in Fig. 5b, the curve fit provides *V*_0_ = 0.65 mm^3^, *λ* = 0.28 d^−1^, with a good extrapolation accuracy (R^2^ = 0.978). It is to be noted that the liver cavity due to the injection of cells has been included to these data (not in the curve fit) indicated as D3 (blue arrow) with a significant volume around 0.044 cm^3^ and appears totally resorbed in the following acquisition at D7. This is confirmed with the 3D volume rendering, presented in Fig. 5c (at D0) and Fig. 5d (at D3 to D21).

Figure 5c shows the vascular location of the contrast agent immediately after injection, and Fig. 5d disclose the localization of the HCC tumor in comparison with the liver. On the one hand, the contrast-enhanced pattern of liver is obtained by a specific affinity of the nano-droplets towards the liver cells, and on the other hand, this induces a negative contrast on liver-embedded tumors that allows delineating the tumor ROI. In Fig. 5d, the contrast-enhanced liver is presented in yellow, with an opacity deliberately reduced to make visible the tumor location (*i.e*. the ROI location). The ROI is colored in blue at D3 referring to the initial liver cavity and then colored in green when it corresponds to the tumor growth (D7 to D21).

**Figure 6:**
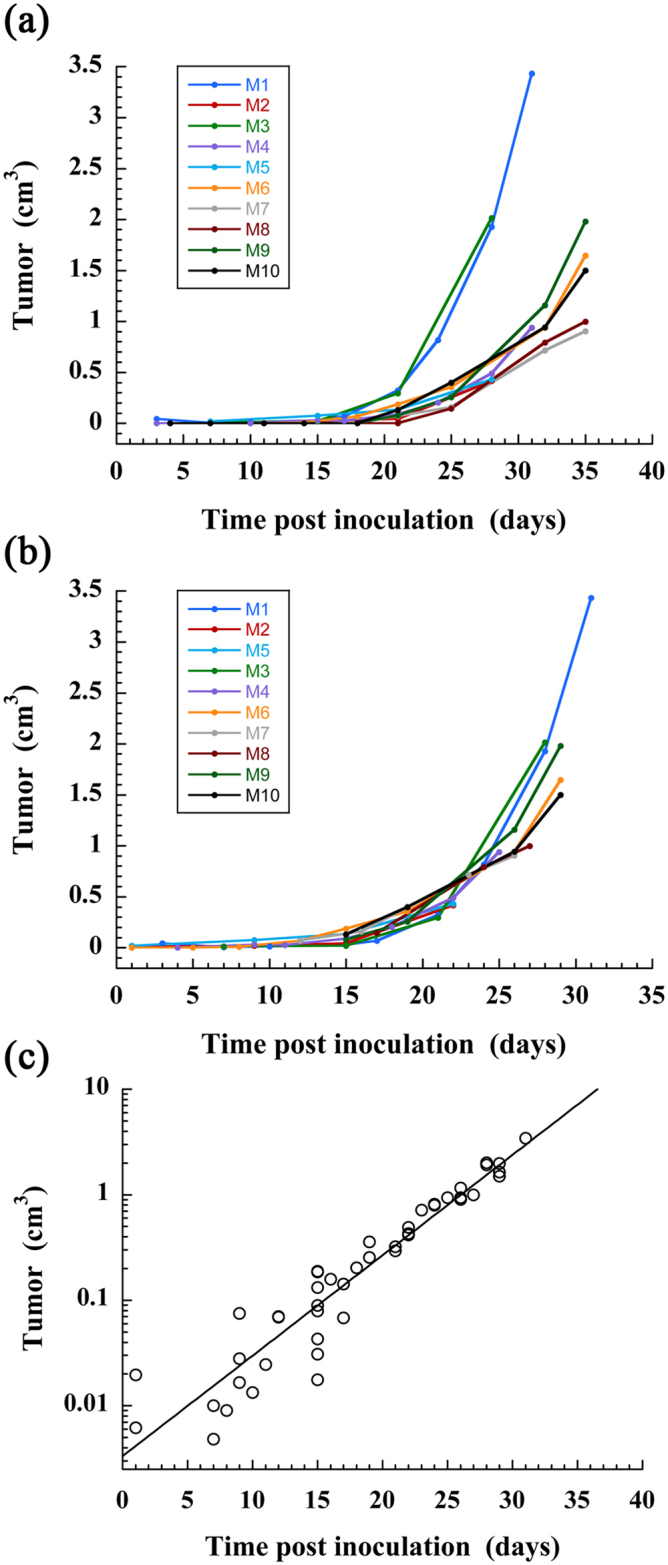
Quantitative follow-up of the tumor volume for a population of mice (n = 10) on which was performed intrahepatic injection of Huh-7 cells, and i.v. administration of 2-3-5-triiodo α-tocopheryl nano-emulsions at D0. (**a**) Raw data. (**b**) Establishment of the master-curve by shifting the data along the time-axis (x-axis), taking M1 as reference. (**c**) Extrapolation of the master-curve using the tumor growth exponential model of Eq. 1.

The same procedure was applied to a group of 10 mice for which the tumor engraftment in liver was successfully obtained, and for which the nano-emulsions were administrated immediately after the inoculation of Huh-7 cells in liver (similarly as the first example described above). Logically the tumor growth is subject-dependent and we observed (Fig. 6a) that the curve rising occurs from D15 to D20 with individual variations. In the hypothesis that the tumor growth constant *λ* is subject-independent, the curves were artificially shifted along the x-axis (taking M1 as reference) to obtain the master-curve (Fig. 6b) that effectively confirmed that the growth constant appears subject-independent. Finally gathering all data allowed using the exponential tumor growth model of Eq. 1, giving a good extrapolation with R^2^ = 0.956 over the 10 mice, and providing values of the fit parameters as *V*_0_ = 3.3 mm^3^, *λ* = 0.22 d^−1^.

### Ultrasound (US) imaging

**Figure 7:**
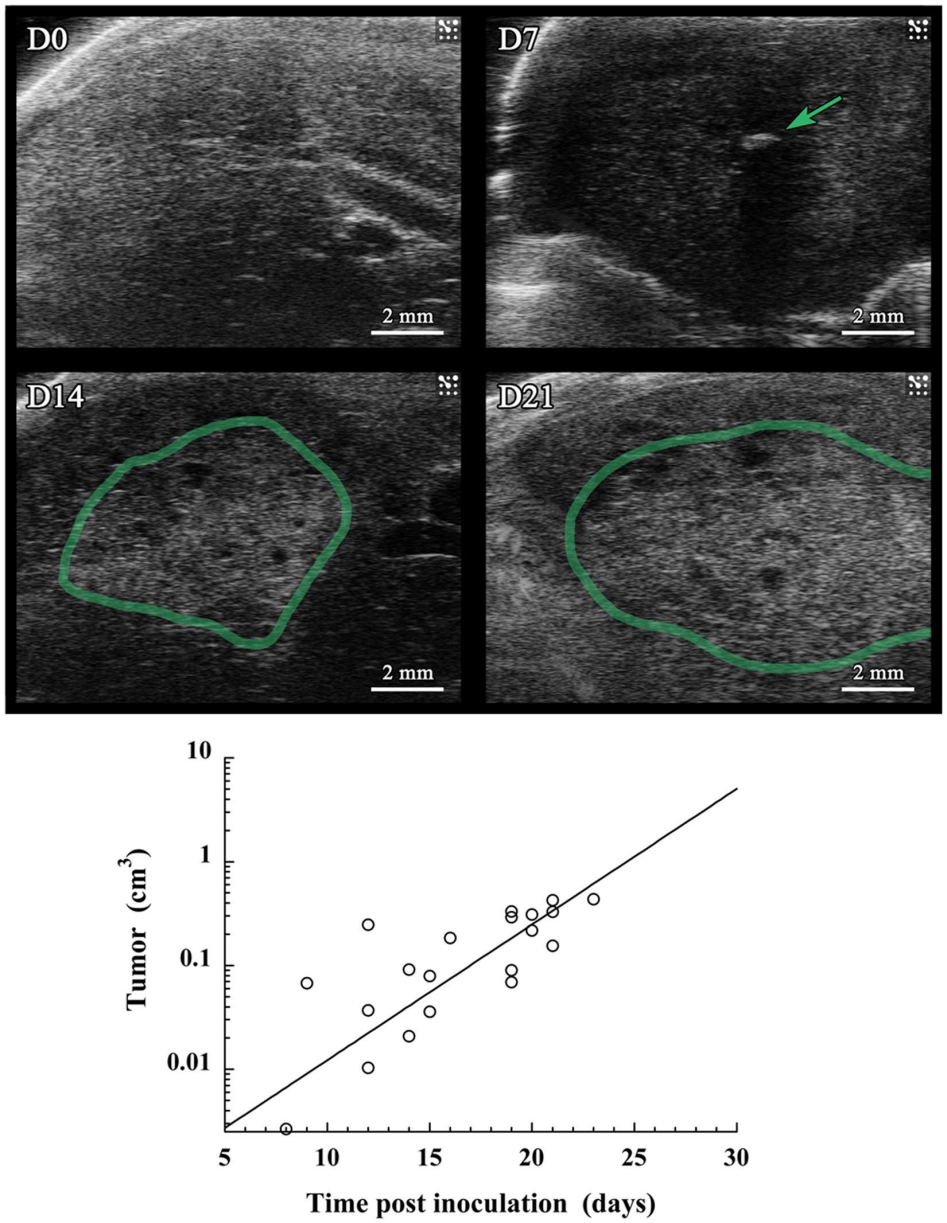
Ultrasound imaging performed on a population of mice (n = 10) on which was performed intrahepatic injection of Huh-7 cells, and i.v. administration of 2-3-5-triiodo α-tocopheryl nano-emulsions at D0. Acquisitions were done at 0, 7, 14 and 21 days post-injection. (**a**) Echography of a representative mouse (M1), on which are indicated in green the tumor region. (**b**) Quantification of the tumor growth through an evaluation of the volume from the echography pictures on the whole population of mice, and using the same *master-curve* based methodology as described in Fig. 6c.

In parallel to the micro-CT acquisitions along the longitudinal study, US imaging (echography) was performed at D0, D7, D14 and D21. The objective of this series of 2D echography experiments was first to confirm the presence of the HCC tumor in liver, and roughly evidence their growth, as a complementary method to micro-CT. Representative images (acquired from M1) are presented in Fig. 7, and shows the absence (D0), the nucleation (D7) and the growth (D14, D21) of the tumor. To compare the sensitivity and the possibilities offered by the two imaging methodologies (echography and the nano-emulsions contrast-enhanced micro-CT), the tumor volume was measured from the echography acquisitions for all the 10 mice and treated per the same methodology followed for Fig. 6c. Volume was estimated according to a rough approximation, considering the tumor regions as spheres, calculating the volume from the diameter as mean of the characteristic dimensions of the tumor. As done before in Fig. 6, master curves were established and were fitted with the similar exponential tumor growth model used (Eq. 1). The data collected for all mice followed are reported in Fig. 7, resulting in a rather poor quality of extrapolation with R^2^ = 0.613, giving values of initial tumor volume as *V*_0_ = 0.60 mm^3^, and a tumor growth constant equal to *λ* = 0.30 d^−1^.

## Discussion

In accordance to the international guidelines and depending on the clinical context, early HCC diagnosis may be performed with a number of imaging modalities, including US, MRI and CT. The contrast agent-free US is most frequently used as a surveillance tool, with MRI and CT as diagnostic options. Herein investigated in preclinical HCC model, this US approach has undoubtedly confirmed the presence of tiny tumor nucleus at D7 (Fig. 7). However, the literature review emphasized as well that the fine quantification of the tumor volume and follow-up of its growth cannot be performed with echography, and is mainly shifted to contrast-enhanced CT and/or MRI, with a limitation in size detection and sensitivity with CT. This limitation is mainly due to the limitation of the existing contrast agents. Our objective was to propose a preclinical evaluation of a novel CT solution to visualize and follow the tumor development with a high sensitivity. Based on preliminary studies on healthy mice previously reported^27,28^, the idea was to prove that the advantage of non-toxic nano-emulsions as CT contrast agents can improve the computed tomography in the detection and analysis of hepatic tumor models. In general, hydrophilic iodinated contrast agents are cleared from the blood and filtered by kidneys within a dozen of minutes, the first advantage of a nanoparticulate system sizing above 10 nm (here distribution centered on 60 nm, see Fig. 1) prevents this first pitfall. In addition, we have previously shown that, besides the PEG decoration of the droplets, the nature of oil induces a strong specificity *in vivo* ^28^, that justifies the choice of 2-3-5-triiodo α-tocopheryl for this liver imaging application, resulting not only in a very high contrast because of the very high iodine concentration, but also in the liver specificity in targeting hepatocytes along with a long hepatic persistence (with *t*_*1/2*_ about 53 days^28^). All these properties of such nano-emulsions exactly coincide with the ones described above for the best MRI contrast agents (*e.g*. T_1_-weighted gadoxetate disodium^17^), and not yet fulfilled and available for CT. Our objective here is to increase the precision of the tools available for CT and micro-CT, generally being *ca*. 30% less precise than the MRI modalities^18–21^.

Expected *in vivo* behavior, pharmacokinetics and liver specificity of the 60 nm 2-3-5-triiodo α-tocopheryl nano-emulsions is confirmed in Figs 2 and 5c and d, nano-emulsions *(i)* acts as blood pool contrast agent at D0 and underwent a total liver accumulation before D3 (doubtlessly from D1), and *(ii)* provide a persistent contrast enhancement in liver up to D24 after a single injection (the persistence being likely much prolonged). The injection cavity in liver presenting a volume of 44 μL (Fig. 5b) is consistent with the volume of Huh-7 cells in culture medium around 50 μL. Unlike US imaging, our methodology allows monitoring the resorption of the injection cavity: still visible at D3, resorbed at D7 (Figs 2 and 5d). Interestingly, the formation of tumor is in the exact place of the initial cavity, as if the bulk phase injected with the cell was removed and the cancerous cells concentrated in one place to form the tumor nucleus (Fig. 2 D7). Then tumor underwent a gradual and exponential growth from D7 to D24 (and after) visible in Fig. 2 and quantified in Fig. 5b. Histopathological investigation confirmed the presence and the location of the tumor structures, as noticed with micro-CT based imaging, and the malignant nature of the differentiated solid hepatocarcinoma, with vascular invasion, cytologic atypia and mitotic activity. Importantly, the good correlation of the experimental data with the mathematical tumor growth model (Fig. 5b), especially for the early point at D7, proves that the volume measured at D7, *i.e*. of 4.8 mm^3^, effectively corresponds to the tumor nucleus and not to the injection cavity. A question raised by Fig. 2 regards the hyper-signal observed in the periphery of the tumor nucleus (at D7) and gradually embedded and engulfed by the tumor itself during its growth. If we consider the morphological details of the HCC along the tumor growth, *e.g*. considering the acquisitions from D15 to D24 of the case reported in Fig. 2, the hyper-signal zone seems to be related to the decrease of the volume occupied by healthy hepatic tissues. In other words, the progression of nodules surrounding the healthy tissue results in confining it and concentrating the contrast agent. Since HCC cells are not able to uptake 2-3-5-triiodo α-tocopheryl (which is revealed in Fig. 4), the iodinated molecules have no other choice to concentrate themselves in confined regions. This is correlated by Fig. 3C (tumor border), showing the compression of normal hepatic cells as a result of the tumor growth, thus logically concentrating the contrast agent, and this is confirmed with a further investigation of the tumor structure presented in the bottom inset of Fig. 2 at D24, detailing transverse sections of tumor for different depths. We can see (from right to left) the different nodules separated from bridges of contrasted healthy liver tissues, which gradually becomes hyper-contrasted. Nevertheless, it is also important to note that this phenomenon has only been observed on subjects developing several tumor nodules that confine healthy tissues as described; some other examples for which only one nodule has grown did not present such hyper-contrasted regions. Therefore, this phenomenon might be used as a surrogate marker of multiple nodules.

An important advantage offered by X-ray imaging, is the possibility to measure directly the concentration of contrast agent in the ROIs since it is linearly proportional to the X-ray attenuation. After normalization of the gray levels measured with air equal to −1000 Hounsfield units (HU) and water equal to 0 HU (for each series of acquisitions, two tubes containing water and air were scanned), computed tomography offers an original tool to follow the biodistribution of contrast agents or X-ray contrasting nano-carriers. This fact allowed above in Fig. 2 to establish that the contrast agent contained in hepatic healthy tissues is not transferred to the tumor during its growth, but can be confined in small domains making increase concentration of contrast agent. This is confirmed with the measurement of X-ray attenuation in liver and tumor, the contrast in liver tissues is high as expected, and the one in the tumor appears very low. However, it should be kept in mind that Huh-7 cells are poorly differentiated cells leading to highly aggressive tumors. More differentiated HCC tumors are usually less aggressive than poorly differentiated HCC tumors. As shown in Figs 2 and 4, unlike healthy liver tissue, the tumor region is not able to keep the iodinated contrast agent. One can easily imagine a relation between high differentiation state and healthy tissues respective to the contrast agent uptake, resulting in staining of the tumor region, which would not be the case with poorly differentiated state. Thus, it should be expected that highly differentiated, poorly aggressive tumors might behave more closely to healthy primary human hepatocytes and, therefore, might be labeled with the contrast agent. If this holds true, one might expect that highly differentiated tumors should be less contrasted with the surrounding liver than poorly differentiated tumors and that the level of contrast between a tumor and the liver parenchyma is an indicator of its aggressiveness. However, this hypothesis should be taken with care since it should also include several confounding factors to consider, including location of the tumor (*e.g*. sub-capsular, peri-hilar), proximity to vessels, background liver disease, tumor size, X-ray parameters and cellular density within the tumor. Further experiments using more differentiated tumor cell lines or different Patient-Derived Xenograft tumor models will be performed to confirm this hypothesis. In addition, both gradually decrease during in function of time. This is the confirmation of the total independence of the tumor growth process towards (and to the detriment of) the healthy liver tissue.

Considering now the whole group of mice followed (Fig. 6), the growth capacity of HCC is logically subject-dependent, probably because, as we recently demonstrated by histology, some cells die during the first few days after injection and only part of the injected cells survive and engraft from D7, leading to the formation of the tumor^32^. However, the methodology undertaken allows emphasizing a growth factor of *λ* = 0.22 d^−1^ common to all mice, and that the difference between mice came from the moment when the tumor begins to growth. The coefficient of determination R^2^ = 0.956 of the exponential curve fit for the whole population, seems to corroborate this hypothesis. The methodology provides an evaluation of the initial tumor volume, *V*_0_ = 3.3 mm^3^, that we could correlate with an estimation of volume of the cells administrated. Retrospectively, this study is not only pioneer because of the precision of the detection and measurements of tumor volume, but also since a preclinical micro-CT-based approach to quantitatively follow the development of tumor in liver has not yet been reported. And this objective has been efficiently reached from the unique properties of the iodinated nano-emulsions, from the concomitance of: *(i)* high iodine loading of the contrast agent that increases the sensitivity, *(ii)* liver and hepatocyte specificity that also contribute to increase the contrast enhancement (and sensitivity), and *(iii)* the strong liver persistence. Another example of literature describing preclinical non-invasive studies that quantitatively follows the growth of tumors in liver by MRI imaging has been reported^29^ using a T_2_-weighted 3D sequence without contrast agent, and confirms the potentials and sensitivity of MRI as exploited in clinic, but also shows that the X-ray imaging (thanks to liver-targeted iodinated nano-emulsions herein described) can effectively reach the standards to MRI in term of sensitiveness, and can even surpass it in term of precision of the volume quantification and visualization of the morphologies of the tumor nodules. The other reported methods for following-up the tumor growth *in vivo* are classically based on bioluminescence (BLI)^30–32^. This approach is important for preclinical research, and leads to a valuable evaluation of the tumor burden and the potential response to therapy, but can encounter some limitations such as a misevaluation of the real volume of the tumor due to scattering and tumor location, and the necessity to implant luminescent tumor. In the case of BLI, the tumor cells themselves provide the fluorescent signal, whereas for MRI or CT, signal comes from contrast agents. Thus, a potential transposition to clinic, in absolute, could be possible for preclinical MRI and CT, potentially through an optimization of the contrast agents, but it is not possible with BLI. US imaging reported in Fig. 7 is another illustration of the complementarity of the different imaging modalities, very efficient to detect the presence of the small tumor nodule at D7, but less sensitive and precise than MRI and now micro-CT about the tumor volume determination and growth follow-up. Applying the same methodology as applied for micro-CT, gives unsatisfactory coefficient of determination R^2^ = 0.613, and gives a growth constant (*λ* = 0.30 d^−1^) far from the reference given by CT in Fig. 6 (*λ* = 0.22 d^−1^).

To conclude, computed tomography is largely widespread and used in clinic because it is a good compromise between simplicity, efficiency and cost, but still in complementarity with MRI, and in general, after the early detection by US imaging. A general disadvantage of CT and micro-CT compared to MRI is the sensitivity of the method, in fact limited by the poor properties of the contrast agents. Here we highlight the strength of 2-3-5-triiodo α-tocopheryl nano-emulsions, as non-toxic, highly X-ray contrasting, liver specific and liver persistent, with properties exactly fitting with the specifications aimed for HCC imaging, or in general tumor imaging, in liver. These nano-emulsions have shown the expected imaging properties for which they were designed and this study opens new research possibilities regarding the role of CT and micro-CT imaging of liver pathologies.

## Methods

### Chemical

2,3,5-Triiodobenzoic acid, α-tocopherol, 4-dimethylaminopyridine, N,N’-dicyclohexylcarbodiimide, dichloromethane, ethyl acetate, cyclohexane solutions were purchased from Sigma-Aldrich, France. Non-ionic surfactant (Kolliphor ELP^®^) from BASF (Ludwigshafen, Germany). Kolliphor ELP^®^ is a parenteral grade nonionic surfactant made by reacting ethylene oxide to castor seed oil at an ethylene oxide to oil molar ratio of 35 (see Fig. 1). Injectable phosphate buffered saline (PBS) were obtained from Eurobio, France.

### Synthesis of 2-3-5-triiodo α-tocopheryl

Iodination grafting on vitamin E was performed per the Steglich reaction previously described^27^. Briefly, α-tocopherol (0.0085 mol, 3.5 g) was dissolved in dichloromethane (DCM) (250 mL). Then the following species were sequentially added, at room temperature, to this DCM phase: 2,3,5-triiodobenzoic acid (5 g, 0.01 mol), 4-dimethylaminopyridine (97 mg, 0.8 mmol) and N,N’-dicyclohexylcarbodiimide (0.11 mol, 2.27 g). The mixture was stirred overnight at room temperature. Dicyclohexylurea and other precipitates were removed by filtration. The organic phase was then sequentially washed with 300 mL distilled water, 300 mL HCl (1 N), 300 mL of a NaHCO_3_ (6 wt.%) solution, 300 mL of saturated brine (NaCl), and finally dried over anhydrous Na_2_SO_4_. The solvent was evaporated and the remaining oil was purified by elution with cyclohexane/ethyl acetate (90/10) over silica gel. Reaction yields were around 80%.

### Formulation and characterization of iodinated nano-emulsions

Iodinated nano-emulsions were formulated spontaneously by low-energy nano-emulsification method^34–36^. Briefly, 0.3 g of 2-3-5-triiodo α-tocopheryl and 0.2 g of Kolliphor ELP^®^ are mixed together in the Thermomixer (Eppendorf) at 2000 rpm and at 80 °C during 1 min, and then the in a sonication bath during ten seconds. This protocol is repeated twice of thrice until the whole mixture is clear, related to the homogeneity of the mixture. In parallel, PBS was heated up also to 80 °C, 750 μL of PBS was added to the former homogeneous phase of 2-3-5-triiodo α-tocopheryl and Kolliphor ELP^®^ that was still maintained at 80 °C. The whole mixture was vortexed for 5 min, giving then stable and monodisperse nano-emulsions sizing around 60 nm (see Fig. 1). Before injection, the samples were sterilized by filtration (0.22 μm membrane, Millex-GP, polyethersulfone (PES) membrane, Millipore, Molsheim, France). Size distributions and polydispersity indices (PDI) were measured by dynamic light scattering (DLS) with a Malvern apparatus (NanoZS, Malvern, Orsay, France). The helium/neon laser, 4 mW, was operated at 633 nm, with the scatter angle fixed at 173° and the temperature maintained at 25 °C. DLS data were analyzed using a cumulants-based method.

### Cell lines

Huh-7 cells (Japanese Collection of Research Bioresources Cell Bank, Osaka, Japan) stably expressing luciferase (Huh-7-Luc) were produced by transduction using a retroviral vector generated from a luciferase-encoding pCLNCX vector (Dr. Lorang, NIH, Bethesda, MD, USA) and cloning by limiting dilution. Huh-7-Luc were cultured in Dulbecco’s Modified Eagle Medium (DMEM) with high glc and Hepes (ref 42430-025, GIBCO, Invitrogen, Cergy Pontoise, France) supplemented with non-essential amino acids (GIBCO^®^), 35 μg/mL gentamycin (Duchefa Biochemie, RV Haarlem, The Netherlands) and 10% fetal bovine serum (PAA, GE Healthcare, Velizy-Villacoublay, France).

### Animal experimentation

Animals were housed in PHENOMIN-ICS licensed animal facility (agreement #B67-218-40). All experiments were assessed by the local ethical committee (Com’Eth, license No. 17) and approved by the French Ministry of Research (Accreditations #4750-2016033116347107). Experiments were performed in accordance with relevant guidelines and regulations. Six-week-old NMRI-nu (Rj:NMRI-Foxn1nu/Foxn1nu) female mice purchased from Janvier Labs (Le Genest Saint Isle, France) were used for experimentation. Huh-7-Luc cells were echo-guided intrahepatic injected according to a procedure previously described^32^. For all injection procedures, the skin of the mice was sterilized with Betadine and ethanol before and after the injection and 1 × 10^6^ Huh-7-Luc cells resuspended in a 50 μL culture medium were injected. Immediately after, iodinated nano-emulsions were administrated i.v. by a retro-orbital injection containing 2-3-5-triiodo α-tocopheryl with an injection volume corresponding to 10% of the blood volume (*i.e*. 7.6 μL of nano-emulsions per gram of mouse). At the end of each manipulation, mice were placed on a heat pad then returned to their regular cage once awakened. A population of 15 mice was used. The injections were performed in the same manner to all participants, and for 10 of them we observed the tumor growth (M1-M10) followed by micro-CT and US, and for the 5 remaining mice (M11-M15) we did not observed any sign of tumor growth during the same period.

### *In vivo* micro-CT imaging

Longitudinal tumor imaging was performed on a total of 15 mice using an *in vivo* micro-CT system (Quantum FX, PerkinElmer, Hopkinton, MA, USA) with the following parameters: 90 kV, 160 µA, 40 × 40 mm field of view and a scan time duration of 4.5 minutes, with respiratory gating. All imaging procedures were carried out under isoflurane (3% for induction and ~1.5% for maintenance) mixed with oxygen (1 L/min) anesthesia. Scans were performed before Huh-7-Luc cells and nano-emulsion injection, immediately after injection, and at 3, 7, 10, 15, 17, 21, 24, 28, 31 and 35 days. Tomographic reconstruction of the projections resulted in 512 slices with an isotropic voxel spacing of 80 µm. Images were converted into DICOM format and analyzed with OsiriX viewer to further establish 2D maximum intensity projection slices, quantify the tumor volume, establish 3D volume rendering images, and quantify the x-ray attenuation by placing the region of interests (ROI) in the liver or tumor.

### *In vivo* ultrasound imaging

Preclinical-grade US imaging was performed at the Mouse Clinical Institute using a preclinical Vevo 2100 echographer and computer (Visualsonics, Tonroto, Ontario, Canada). B-Mode, or brightness mode, imaging is used to acquire two-dimensional images of an area of interest and for identification of anatomical structures.s
